# Genome Sequence and QTL Analyses Using Backcross Recombinant Inbred Lines (BILs) and BILF_1_ Lines Uncover Multiple Heterosis-related Loci

**DOI:** 10.3390/ijms21030780

**Published:** 2020-01-25

**Authors:** Yahui Yu, Mengmeng Zhu, Yue Cui, Yu Liu, Zhenyu Li, Nan Jiang, Zhengjin Xu, Quan Xu, Guomin Sui

**Affiliations:** 1 Rice Research Institute, Shenyang Agricultural University, Shenyang 110866, China; yyh3655@126.com (Y.Y.);; 2 Liaoning Institute of Saline-Alkali Land Utilization, Panjin 124010, China; 3 Liaoning Academy of Agricultural Sciences, Shenyang 110866, China; 4 Shenyang Research and Development Service Center of Modern Agriculture, Shenyang 110034, China

**Keywords:** rice, heterosis, yield components, high-throughput sequence

## Abstract

Heterosis is an interesting topic for both breeders and biologists due to its practical importance and scientific significance. Cultivated rice (*Oryza sativa* L.) consists of two subspecies, *indica* and *japonica*, and hybrid rice is the predominant form of *indica* rice in China. However, the molecular mechanism underlying heterosis in *japonica* remains unclear. The present study determined the genome sequence and conducted quantitative trait locus (QTL) analysis using backcross recombinant inbred lines (BILs) and BILF_1_ lines to uncover the heterosis-related loci for rice yield increase under a *japonica* genetic background. The BIL population was derived from an admixture variety Habataki and *japonica* variety Sasanishiki cross to improve the genetic diversity but maintain the genetic background close to *japonica*. The results showed that heterosis in F_1_ mainly involved grain number per panicle. The BILF_1_s showed an increase in grain number per panicle but a decrease in plant height compared with the BILs. Genetic analysis then identified eight QTLs for heterosis in the BILF_1_s; four QTLs were detected exclusively in the BILF_1_ population only, presenting a mode of dominance or super-dominance in the heterozygotes. An additional four loci overlapped with QTLs detected in the BIL population, and we found that *Grains Height Date 7* (*Ghd7*) was correlated in days to heading in both BILs and BILF_1_s. The admixture genetic background of Habataki was also determined by subspecies-specific single nucleotide polymorphisms (SNPs). This investigation highlights the importance of high-throughput sequencing to elucidate the molecular mechanism of heterosis and provides useful germplasms for the application of heterosis in *japonica* rice production.

## 1. Introduction

Food security is a major global problem as the competition for arable land between food and energy crops, and population growth, continue to increase. Improving crop productivity has been the key focus of national and international efforts in breeding crops such as maize and rice. The vigor of *indica* rice hybrids often exhibits phenotypes that surpass their parents in terms of growth and fertility, which is also known as heterosis. In crop production, successful agronomic exploitation of yield heterosis has been achieved in the past decades. Three major competing but non-mutually-exclusive hypotheses (dominance, overdominance, and epistasis) have been proposed to explain heterosis at the genetic level [1,2,3,4,5,6]. However, the progress in elucidating the molecular mechanism underlying crop heterosis has lagged.

In the past decades, heterosis has become a priority research target for both breeders and scientists based on its practical importance and scientific significance. Heterosis in crop breeding was first applied to hybrid maize in the 1930s, and the three-line method or cytoplasmic male sterility (CMS) system contributed to the commercialization of hybrid rice in the 1970s. Crop heterosis has been extensively applied to rice and maize production, significantly improving global yield compared with traditional inbred lines [7]. Currently, the planting area of hybrid rice accounts for 50–60% of the total rice planting area, and about 80% of *indica* rice is hybrid and is mainly planted in southern China. A study used recombinant inbred lines (RIL) derived from a cross between PA64s and 93-11, and RIL backcross F_1_ populations were analyzed to elucidate the molecular mechanism of heterosis among *indica* and *javanica* varieties [8]. A mega sequence project for 10,0742 F_2_ lines revealed the genomic architecture of heterosis for yield traits in rice using the cross combination of *Oryza sativa* subspecies (ssp.) *indica*–*indica* (three-line system), *indica*–*indica* (two-line system), and *O. sativa* ssp. *indica*–*O. sativa* ssp. *japonica* crosses [9]. Nevertheless, research on heterosis in *japonica* is limited. Almost all *japonica* varieties are conventional rice, and the hybrid *japonica* accounts for less than 3% among total *japonica*, which is mainly distributed in northern China and the middle range of China. The disappearance of hybrid *japonica* rice is due to the lack of genetic diversity among *japonica* cultivars.

In this study, we used a backcross RIL derived from the cross between Habataki (an admixture variety between the *indica* and *japonica* variety) and *japonica* variety Sasanishiki, and F_1_ plants were backcrossed to Sasanishiki once before inbreeding. The backcross improved genetic diversity through the introgression of the *indica* pedigree while maintaining the population under the *japonica* genetic background. After inbreeding for 10 generations, we obtained 85 lines of the backcross recombinant inbred line (BIL) population. Then, we crossed all of the 85 lines to Sasanishiki again to generate the BILF_1_ population. The present study used genome sequencing and quantitative trait locus (QTL) analysis of BILs and BILF_1_s to identify heterosis-related loci for yield increase.

## 2. Results

### 2.1. Population Sequencing and Linkage Map Construction

**Figure 1:**
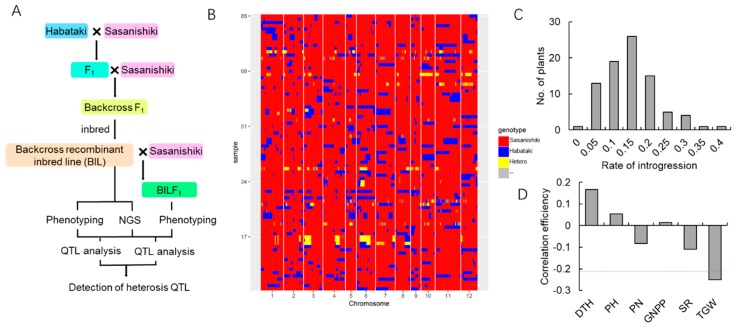
Schematic overview of the parent backcross recombinant inbred line (BIL) system construction and the map of genome-wide graphic genotypes. (**A**) The technology roadmap using genome sequence and quantitative trait locus (QTL) analysis of BILs and BILF_1_s to uncover heterosis-related loci for yield increase. (**B**) Graphic genotypes of 85 BILs were identified by a sliding window approach along each chromosome. Various colors represent different genotypes. (**C**) The introgression rate of the Habataki pedigree among the BILs. (**D**) The correlation between the introgression rate of the Habataki pedigree and yield-related traits. The dotted lines indicate significance at the 5% level.

We used a strategy of sequencing-based map construction to conduct QTL mapping for the BIL derived from the cross between Sasanishiki and Habataki (Figure 1A). We sequenced a segregating population of Sasanishiki and Habataki BILs together with parental lines on an Illumina HiSeq2500 platform. A total of 224.02 GB of raw data were generated for all of the BILs, with approximately 6.29-fold depth for each BIL, 22.94 GB for Habataki (51.00-fold), and 23.24 GB for Sasanishiki (56.00-fold). We aligned the sequence data to the reference genome (Os-Nipponbare-Reference-IRGSP-1.0) using SOAP2 software [10,11]. A total of 1,947,668 single-nucleotide polymorphisms (SNPs) with homozygous genotypes between both parents were identified using the SOAPsnp software [12]. These SNPs were used as potential markers in the subsequent analysis. The SNP markers localized to highly repetitive regions, and those with low genotyping scores were removed to avoid ambiguity in linkage map construction. We used an effective imputation model, k-nearest neighbor algorithm to impute the missing genotypes of each RIL caused by low-coverage sequencing [13]. Finally, we used 1,591,495 high-quality polymorphic SNP markers to construct a recombinant bin map (Figure 1B). Subsequently, a recombinant bin map was constructed, and the map contained 3652 recombinant blocks, with the average genetic length of 0.44 cM. Then, we determined the introgression rate of the Habataki pedigree using the data of bin map. The introgression rate showed a normal distribution that indicated that the population is ideal for the subsequent survey (Figure 1C). We analyzed the correlation between the introgression rate of Habataki and the important yield-related traits. The results showed that the introgression rate was significantly negatively correlated to 1000-grain weight (TGW) (Figure 1D).

### 2.2. Grain Yield Heterosis is the Result of Hybrid Vigor in Grain Number Per Panicle

**Figure 2:**
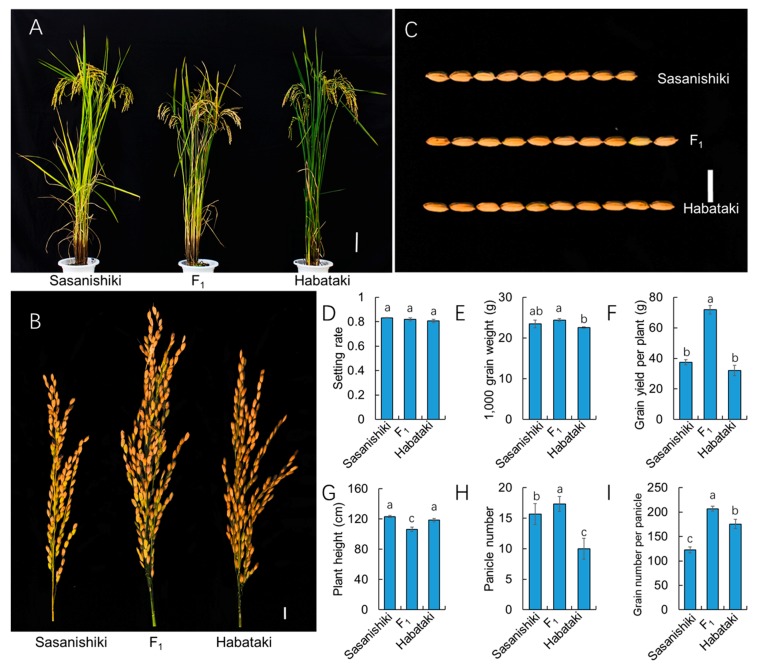
Heterosis in F_1_ plants. (**A**) The plant architecture of F_1_ plant and parental lines. Scale bar = 10 cm. (**B**) The panicle of F_1_ plants and parental lines. Scale bar = 1 cm. (**C**) Grains of F_1_ plants and parental lines. Scale bar = 10 cm. (**D**–**I**) the yield-related traits of F_1_ plants and parental lines.

To obtain ideal agronomic characteristics of heterosis, the paternal line itself is usually an excellent inbred variety with superior agronomic performance. In addition, the hybrid F_1_ plant should surpass its paternal parent, particularly the traits related to yield components. Interestingly, the F_1_ plants did not exhibit greater plant height compared with the parental lines in the present study (Figure 2A). We surveyed the yield components of the parental lines and F_1_ plants (Figure 2). The panicle length of the F_1_ plants was significantly longer than that of Sasanishiki and Habataki (Figure 2B). Sasanishiki exhibited a short round grain shape, whereas the F_1_ and Habataki exhibited a slender grain shape. However, the 1000-grain weight of Habataki was significantly lower than that of the F_1_ plants, whereas the 1000-grain weight of Sasanishiki was similar to that of the F_1_ plants. The results showed that the F_1_ plants exhibited an advantage in grain number per panicle and panicle number compared with Sasanishiki and Habataki. A similar setting rate was observed in F_1_, Sasanishiki, and Habataki. The slenderest grain shape of Habataki had the lowest 1000-grain weight in the parent line and F_1_ plants, whereas Sasanishiki had similar 1000-grain weight as the F_1_ plants. Taken together, the advantage in panicle number and grain number per panicle makes the F_1_ plants exhibit heterosis in grain yield per plant.

### 2.3. Yield Components and Other Important Agronomic Traits of BILs and BILF_1_s

**Figure 3:**
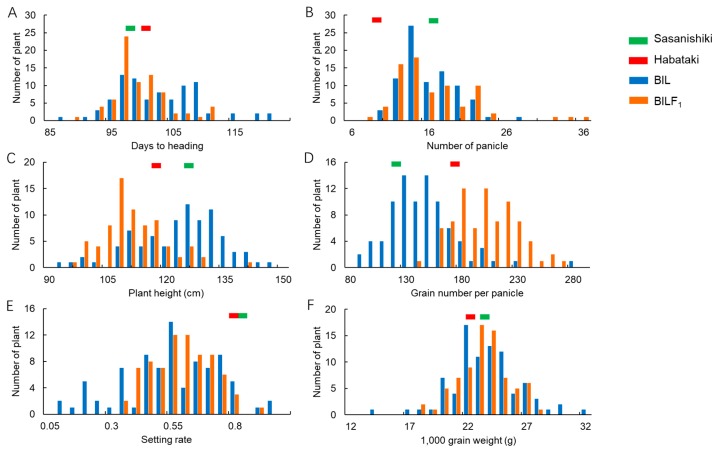
The distribution of yield-related traits in BILs and BILs. (**A**–**F**) The distribution of days to heading, number of panicles, plant height, grain number per panicle, setting rate, and 1000-grain weight in BILs and BILF_1_s.

To assess the distribution pattern of yield-related traits between BILs and BILF_1_s, we surveyed the yield components of BILs and BILF_1_s in the paddy field of Shenyang Agricultural University in 2018. The normal distribution and transgressive segregation were observed in all of the traits surveyed in both BIL and BILF_1_s. These results indicate that all of these agronomic traits were controlled by multiple genes. The plant height of BILF_1_s mainly ranged from 105 to 120 cm, whereas that of BILs was from 120 to 135 cm. These results indicate that the heterozygous genotype of the F_1_ plants reduces the plant height, based on the fact that the F_1_ plants were shorter than Sasanishiki and Habataki (Figure 1A). In addition, the grain number per panicle of BILs ranged from 80 to 180, whereas that of the BILF_1_s ranged from 130 to 260. These results suggest that the heterozygous genotype of the F_1_ plants increases the grain number per panicle, which also coincides with the result that the F_1_ plants have significantly more grains per panicle than Sasanishiki and Habataki (Figure 2). Similar distributions of days to heading, panicle number, and 1000-grain weight were observed in both BILs and BILF_1_s. However, the variation range of setting rate in BILs was larger than in the BILF_1_s (Figure 3).

### 2.4. QTL Detection and Analysis Using the BILs and BILF_1_ Population

**Figure 4:**
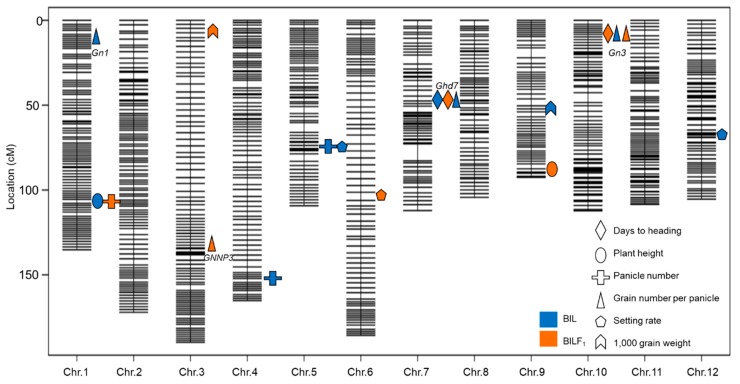
The position of quantitative trait locuses (QTLs) for yield-related traits and heterosis QTLs in BILs and BILF_1_s. Different colors represent the QTLs detected in BILs and BILF_1_s.

**Figure 5:**
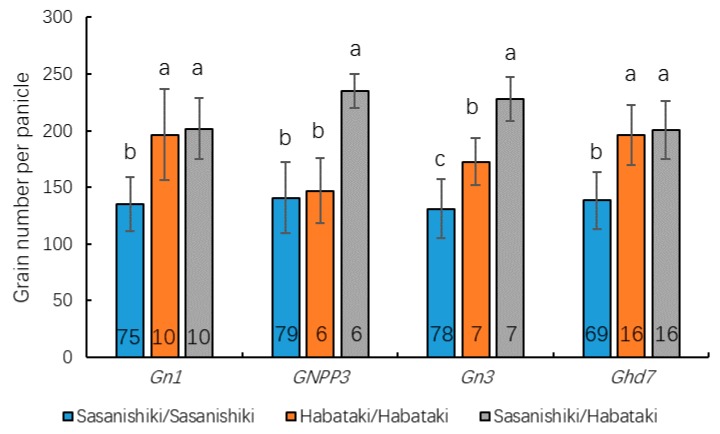
Grain number per panicle of each genotype for the four QTLs detected in BIL and BILF_1s_. The number inside the column represents the number of plants for each genotype. All data are given as mean ± s.e.m.,different letters indicate the significant differences at the 5% level.

To elucidate the genetic mechanism underlying heterosis of yield traits, we primarily focused on the days to heading (DTH), plant height (PH), panicle number (PN), grain number per panicle (GNPP), setting rate (SR), and 1000-grain weight (TGW). A molecular linkage map with 3652 bins was constructed based on sequence variations in Sasanishiki and Habataki and those among the BILs using Highmaps software. In the BIL population, 10 QTLs for all of the traits were mapped independently to rice chromosomes 1, 4, 5, 7, 9, 10, and 12 (Figure 4). To detect QTLs for heterosis that was associated with the effects of the heterozygous on the genetic background of Sasanishiki, we used the BILF_1_ to conduct QTL analysis. Eight loci for the respective phenotypes were detected on chromosomes 1, 3, 6, 7, 9, and 10. Among the eight QTLs, four of these loci overlapped with QTLs detected in the BIL population, and four QTLs were detected independently in the BILF_1_ population. These results presented a mode of dominance or super-dominance in the heterozygote. Among the QTLs detected in both BILs and BILF_1S_, the cluster at the short arm of chromosome 10 corresponded to the days to heading in BILF_1_s and the grain number per panicle in both BILs and BILF_1_s. In addition, a cluster at chromosome 7 was related to days to heading in both BILs and BILF_1_s. As heterosis was mainly observed in the grain number per panicle, we subsequently summarized the grain number per panicle of each genotype for the four QTLs detected in the BIL and BILF_1s_ (Figure 5). The results showed that in the *Gn1* and *Ghd7* loci, the heterozygous genotype and the homozygous Habataki genotype plants had significant advantage in grain number per panicle compared with the homozygous Sasanishiki genotype plants. In the *GNNP3* locus, the homozygous Sasanishiki and homozygous Habataki genotype plants had similar grain number per panicle but significantly fewer than the heterozygous genotype plants. In the *Gn3* locus, the heterozygous genotype plants showed a significant increase of grain number per panicle compared with homozygous Habataki genotype plants, whereas the homozygous Habataki genotype plants had a significantly higher grain number per panicle than the homozygous Sasanishiki genotype plants.

### 2.5. The Indica/Japonica Pedigree Analysis of Sasanishiki and Habataki

**Figure 6:**
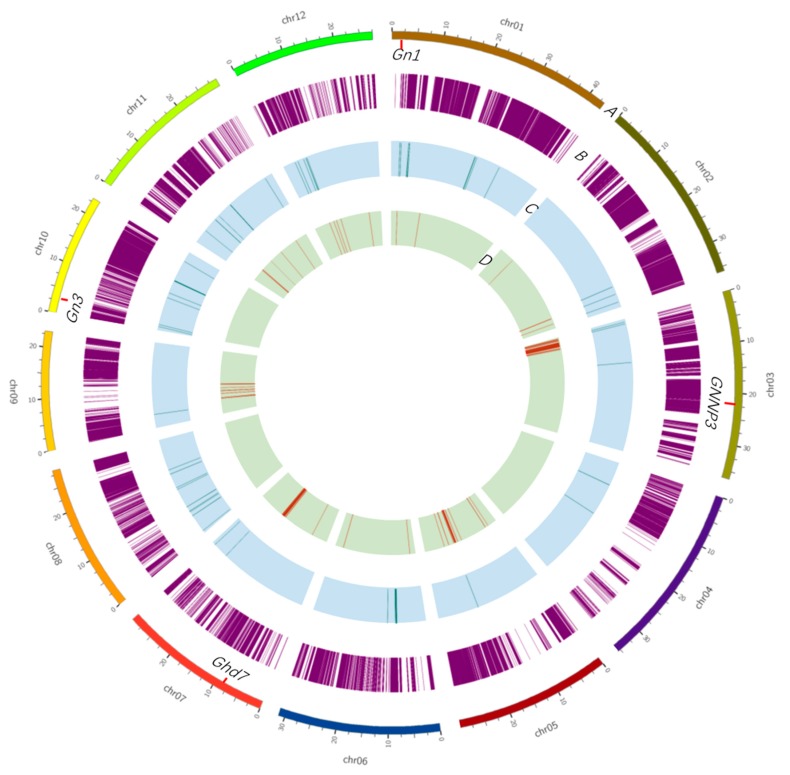
Overview of the subspecies-specific SNPs and introgression in Sasanishiki and Habataki. Tracks from the outer to inner circles indicate the following. (**A**) The chromosome length. The loci of grain number per panicle are indicated on the inside of the circle. (**B**) The distribution of 81,690 subspecies-specific SNPs in the genome. (**C**) The introgression of *japonica*-type SNPs into the genome of Habataki. (**D**) The introgression of *indica*-type SNPs in the genome of Sasnishiki.

Breeding history indicates that Habataki is an admixture line between *indica* and *japonica*. Thus we conducted an analysis of the *indica*/*japonica* pedigree of Sasanishiki and Habataki. The *indica*/*japonica* pedigree was defined using the subspecies-specific SNPs. The subspecies-specific SNPs were those of the same type in all *japonica*, but not in *indica*, which is based on the divergence of the 517 rice landraces [14]. In total, 100,529 subspecies-specific SNPs were selected. We matched the 1,947,668 SNPs between Sasanishiki and Habataki to 100,529 subspecies-specific SNPs, and 81,690 SNPs were merged. The 81,690 SNPs were then used to analyze the *indica*/*japonica* pedigree of Sasanishiki and Habataki. The results showed that there are 621 *japonica*-type SNPs in the genome of Habataki, which indicated that 0.76% of *japonica* genomic introgression involved Habataki. Meanwhile, there were only 81 *indica*-type SNPs in Sasanishiki, indicating that only 0.01% *indica* pedigree introgression occurred in the Sasanishiki genome. The distribution of subspecies-specific SNPs and *indica*/*japonica* genomic introgression is shown in Figure 6. Agronomic-traits-related QTLs were located out of the *indica*/*japonica* genomic introgression, which confirmed that the heterosis QTL originated from the difference between *indica* and *japonica*.

## 3. Discussion

Since its generation in 1973, hybrid rice has predominated *indica* rice production in China. Numbers of elite combinations have been developed and released as commercial varieties, with yields roughly 20% higher than their inbred counterparts [15]. Recent molecular research has investigated the number of hybrid combinations to construct a model system for studying the molecular mechanism of heterosis for three- and two-line hybrids. A study using the yield components data and an ultra-high-density SNP bin map of an immortalized F_2_ population derived from the cross between Zhenshan97 and Minghui63 demonstrated that the relative contributions of the genetic components vary with traits. The results indicate that overdominance/pseudo-overdominance are most important to the heterosis of grain number per panicle, 1000-grain weight, and grain yield per plant. In heterosis of panicle number and 1000-grain weight, the dominance × dominance interaction is important. Among these yield-related traits, single-locus dominance has relatively small contributions [16]. An integrated analysis using the RIL population and RILBCF_1_ population derived from the cross between PA64S (which has a mix genetic background of *indica* and *javanica*) and 93-11 showed that heterosis was mainly detected in grain number per panicle and panicle number [8]. Huang et al. sequenced 10,074 F_2_ lines derived from 17 representative hybrid combinations, and they found that a small number of genomic loci from female parents explain a large proportion of the yield advantage of hybrids over their male parents [9]. Taken together, these studies improve our understanding of heterosis. However, research on heterosis between *japonica* and *japonica* crosses is limited. The genetic diversity of *japonica* was not as high as that of *indica*, making heterosis among *japonica* inconspicuous compared with that among *indica*. Moreover, several factors have limited the utilization of heterosis between *indica* and *japonica*, which includes sterility. Thus, we used a BIL population derived from an *indica* and *japonica* cross to improve the genetic diversity but maintain the genetic background close to *japonica*. The results of the present study showed that heterosis between Sasanishiki and Habataki mainly came from a complicated quantitative and components-specific phenotype. Here, we clearly demonstrate yield heterosis, mainly by the outperformance of grain number per panicle and panicle number. Yield components survey also showed that the grain number per panicle of BILF_1_ population was significantly higher than that of the BIL population. Our previous study demonstrated that the introgression of *indica* pedigree in the *japonica* genome contributed to the increase of rice production in northern China [17], and thus the present study confirmed that the *indica* pedigree could increase the grain number per panicle in *japonica*.

Using high-throughput sequencing, we conducted QTL mapping of the BIL and BILF_1_ populations, and the number of QTLs that are responsible for yield and yield-related heterosis was determined. A total of 10 QTLs for all of the traits were mapped independently in BIL population (Figure 4), and eight loci for the respective phenotypes were detected. Among the eight QTLs, four of these loci overlapped with QTLs detected in the BIL population, and the remaining four QTLs were detected exclusively in the BILF_1_ population, indicating a mode of dominance or super-dominance in the heterozygote. At the gene level, Gao et al. demonstrated that a heading-time-regulated gene *Day to Heading 8 (DTH8)* is a candidate locus for yield heterosis in Liang-you-pei 9 (LYP9) [18], and Li et al. and Huang et al. confirmed that *DTH8* corresponds to yield heterosis [8,9]. These results suggest that the heading time gene strongly participates in yield heterosis in hybrid rice. The present study detected that the heading-time gene *Ghd7* is also a candidate locus for both yield heterosis and heading time regulation. A study using the BILs derived from the cross between Habataki and Koshihikari identified five QTLs (*Gn1*–*Gn5*) that were related to grain number per panicle on chromosome 1, 4, 10, and 12 [19]. The present study confirmed that *Gn1* and *Gn3* correspond to grain number per panicle. Moreover, *Gn3,* on the short arm of chromosome 10, also corresponded to grain number per panicle in BILF_1_s. Thus, this QTL may be further assessed in terms of heterosis.

## 4. Materials and Methods

### 4.1. Plant Materials

The parental line Sasanishiki is typical *japonica* varieties, and the parental line Habataki is an admixture variety with both *indica* and *japonica* pedigree. Sasanishiki was crossed to Habataki, and the F_1_ plant was crossed to Sasanishiki, and then inbred over 10 generations by single-seed descent to generate a population containing 85 BILs. The BILs were then backcrossed to the maternal parent Sasanishiki to obtain 85 BILF_1_s. All materials are maintained at the Rice Research Institute of Shenyang Agricultural University (Shenyang, China). All plants were planted in random block design under standard agricultural management practice in the paddy field of Shenyang Agricultural University (N41°, E123°). All lines were planted with three biological replicates A total of 300 plants in a 16 m^2^ plot were characterized for each line. Plant height, panicle number, grain number per panicle, setting rate, and 1000-grain weight were surveyed in the field in 2018. Both BILs and BILF_1_s were used in QTL analysis.

### 4.2. DNA Extraction and QTL Analysis

Young leaves of each line were collected two weeks after transplant. The CTAB method was used in extracting the high-quality genomic DNA. The sequencing libraries were constructed on an Illumina HiSeq2500 platform (Illumina, Inc.; San Diego, CA, USA) according to the manufacturer’s instructions. We aligned the sequence data to the reference genome (Nipponbare, http://rapdb.dna.affrc.go.jp/download/irgsp1.html/) using SOAP2 software [11]. To construct the genetic linkage map for QTL analysis, we combined the cosegregating SNP/InDel into bins via HighMap software [20]. A map containing 3652 bins and 1592.12 cM in length was constructed with an average of 304 bins on each chromosome. Twelve linkage groups corresponded to the 12 rice chromosomes. We observed the full collinearity between the genetic map and the rice genome, and the minimum value of the Spearman coefficient for chromosomes was 0.962 (Chr. 7). The HighMap software was used to construct a linkage map. The software constructs high-quality linkage map according to the maximum likelihood estimation method. We used the R/qtl (version: 1.44-9) software to conduct QTL analysis via a composite interval mapping (CIM) model. The significance thresholds were determined by 1000 permutations. The percentage of phenotypic variance calculation explained by each QTL was obtained according to the population variance within the mapping population. The details of the QTL analysis were described in our previous studies [21].

## References

[1] Fiévet J.B., Thibault N., Christine D., de Dominique V. (2018). Heterosis Is a Systemic Property Emerging From Non-linear Genotype-Phenotype Relationships: Evidence From in Vitro Genetics and Computer Simulations. Front. Genet..

[2] Bruce B.A. (1910). The Mendelian Theory of Heredity and the Augmentation of Vigor. Science.

[3] Jones D.F. (1917). Dominance of Linked Factors as a Means of Accounting for Heterosis. Proc. Natl. Acad. Sci. USA.

[4] Shull G.H. (1911). The Genotypes of Maize. Am. Nat..

[5] East E.M. (1936). Heterosis. Genetics.

[6] Minvielle F. (1987). Dominance is not necessary for heterosis: A two-locus model. Genet. Res..

[7] Xu Y. (2010). Developing Marker-Assisted Selection Strategies for Breeding Hybrid Rice.

[8] Li D., Huang Z., Song S., Xin Y., Mao D., Lv Q., Zhou M., Tian D., Tang M., Wu Q. (2016). Integrated analysis of phenome, genome, and transcriptome of hybrid rice uncovered multiple heterosis-related loci for yield increase. Proc. Natl. Acad. Sci. USA.

[9] Huang X., Yang S., Gong J., Zhao Q., Feng Q., Zhan Q., Zhao Y., Li W., Cheng B., Xia J. (2016). Genomic architecture of heterosis for yield traits in rice. Nature.

[10] Kawahara Y., Bastide M.D.L., Hamilton J.P., Kanamori H., Mccombie W.R., Shu O., Schwartz D.C., Tanaka T., Wu J., Zhou S. (2013). Improvement of the Oryza sativa Nipponbare reference genome using next generation sequence and optical map data. Rice.

[11] Li R., Yu C., Li Y., Lam T.W., Yiu S.M., Kristiansen K., Wang J. (2009). SOAP2: An improved ultrafast tool for short read alignment. Bioinformatics.

[12] Li R., Li Y., Fang X., Yang H., Wang J., Kristiansen K., Wang J. (2009). SNP detection for massively parallel whole-genome resequencing. Genome Res..

[13] Huang X., Wei X., Sang T., Zhao Q., Feng Q., Zhao Y., Li C., Zhu C., Lu T., Zhang Z. (2010). Genome-wide association studies of 14 agronomic traits in rice landraces. Nat. Genet..

[14] Huang X., Zhao Y., Wei X., Li C., Wang A., Zhao Q., Li W., Guo Y., Deng L., Zhu C. (2012). Genome-wide association study of flowering time and grain yield traits in a worldwide collection of rice germplasm. Nat. Genet..

[15] Pan X.W., Qiang H.E., Zhang W.H., Shu F., Xing J.J., Sun P.Y., Deng H.F., University H.A. (2015). Reflection of Rice Development in Yangtze River Basin under the New Situation. Hybrid Rice.

[16] Zhou G., Chen Y., Yao W., Zhang C., Xie W., Hua J., Xing Y., Xiao J., Zhang Q. (2012). Genetic composition of yield heterosis in an elite rice hybrid. Proc. Natl. Acad. Sci. USA.

[17] Sun J., Liu D., Wang J.-Y., Ma D.-R., Tang L., Gao H., Xu Z.-J., Chen W.-F. (2012). The contribution of intersubspecific hybridization to the breeding of super-high-yielding japonica rice in northeast China. Theor. Appl. Genet..

[18] Gao Z.-Y., Zhao S.-C., He W.-M., Guo L.-B., Peng Y.-L., Wang J.-J., Guo X.-S., Zhang X.-M., Rao Y.-C., Zhang C. (2013). Dissecting yield-associated loci in super hybrid rice by resequencing recombinant inbred lines and improving parental genome sequences. Proc. Natl. Acad. Sci. USA.

[19] Ashikari M., Sakakibara H., Lin S., Yamamoto T., Takashi T., Nishimura A., Angeles E.R., Qian Q., Kitano H., Matsuoka M. (2005). Cytokinin oxidase regulates rice grain production. Science.

[20] Liu D., Ma C., Hong W., Huang L., Liu M., Liu H., Zeng H., Deng D., Xin H., Song J. (2014). Construction and Analysis of High-Density Linkage Map Using High-Throughput Sequencing Data. PLoS ONE.

[21] Li X., Wu L., Wang J., Sun J., Xia X., Geng X., Wang X., Xu Z., Xu Q. (2018). Genome sequencing of rice subspecies and genetic analysis of recombinant lines reveals regional yield- and quality-associated loci. BMC Biol..

